# A CT texture-based nomogram for predicting futile reperfusion in patients with intraparenchymal hyperdensity after endovascular thrombectomy for acute anterior circulation large vessel occlusion

**DOI:** 10.3389/fneur.2024.1327585

**Published:** 2024-04-19

**Authors:** Meijuan Dong, Chun Chen, Wei Chen, Kun An

**Affiliations:** ^1^Department of Endocrinology, The Affiliated Huaian No.1 People′s Hospital of Nanjing Medical University, Huai'an, China; ^2^Department of Neurology, Xuzhou Medical University Affiliated Hospital of Huai’an, Huai'an, China; ^3^Department of Radiology, The Affiliated Huaian No.1 People′s Hospital of Nanjing Medical University, Huai'an, China; ^4^Department of Neurology, The Affiliated Huaian No.1 People′s Hospital of Nanjing Medical University, Huai'an, China

**Keywords:** acute ischemic stroke, endovascular thrombectomy, intraparenchymal hyperdensity, futile reperfusion, nomogram, CT texture

## Abstract

**Background:**

Post-thrombectomy intraparenchymal hyperdensity (PTIH) in patients with acute anterior circulation large vessel occlusion is a common CT sign associated with a higher incidence of futile reperfusion (FR). We aimed to develop a nomogram to predict FR specifically in patients with PTIH.

**Methods:**

We retrospectively collected information on patients with acute ischemic stroke who underwent endovascular thrombectomy (EVT) at two stroke centers. A total of 398 patients with PTIH were included to develop and validate the nomogram, including 214 patients in the development cohort, 92 patients in the internal validation cohort and 92 patients in the external validation cohort. The nomogram was developed according to the independent predictors obtained from multivariate logistic regression analysis, including clinical factors and CT texture features extracted from hyperdense areas on CT images within half an hour after EVT. The performance of the nomogram was evaluated with integrated discrimination improvement (IDI), category-free net reclassification improvement (NRI), the area under the receiver operating characteristic curve (AUC-ROC), calibration plots, and decision curve analyses for discrimination, calibration ability, and clinical net benefits, respectively.

**Results:**

Our nomogram was constructed based on three clinical factors (age, NIHSS score and ASPECT score) and two CT texture features (entropy and kurtosis), with AUC-ROC of 0.900, 0.897, and 0.870 in the development, internal validation, and external validation cohorts, respectively. NRI and IDI further validated the superior predictive ability of the nomogram compared to the clinical model. The calibration plot revealed good consistency between the predicted and the actual outcome. The decision curve indicated good positive net benefit and clinical validity of the nomogram.

**Conclusion:**

The nomogram enables clinicians to accurately predict FR specifically in patients with PTIH within half an hour after EVT and helps to formulate more appropriate treatment plans in the early post-EVT period.

## 1 Introduction

Acute ischemic stroke (AIS) is the second leading cause of death and disability worldwide (1). Endovascular thrombectomy (EVT), which has been shown to be the primary therapy for AIS due to large vessel occlusion, significantly improves functional outcome and reduces mortality (2). Nonetheless, a considerable number of patients (41–55%) treated with EVT failed to achieve a favorable outcome at 3 months, despite successful reperfusion, which is known as futile reperfusion (FR) (3).

Post-thrombectomy intraparenchymal hyperdensity (PTIH) is a common CT sign, with an incidence of 31%–84% in previous studies (4), and is associated with increased mortality and worse clinical outcome (4, 5). Therefore, we should pay more attention to patients with PTIH after EVT. The presence of PTIH indicates successful reperfusion but is inevitably accompanied by reperfusion injury. Notably, the efficacy of reperfusion after EVT in patients with PTIH varied widely with different degrees of reperfusion injury, making it difficult for clinicians to accurately predict the efficacy of reperfusion after EVT in these patients at an early stage. Therefore, there is an urgent requirement to accurately stratify the risk of FR in patients with PTIH and to offer further intervention after EVT to high-risk patients to optimize their prognosis. However, prognostic models specific to patients with PTIH are lacking.

Radiomics is the quantitative analysis of medical images that can be used to accurately and non-invasively diagnose and predict prognosis (6). CT Texture Analysis (CTTA), a part of radiomics, is based on texture features of CT images to extract the wealth of information hidden inside CT images (7). There is emerging evidence that radiomics may be useful in predicting functional outcome at 3 months in patients with AIS. Radiomic features extracted from infarct lesions on diffusion-weighted imaging (DWI) showed good performance in predicting functional outcome in patients with AIS (8). CTTA with early ischemic CT signs as the a region of interest (ROI) was recently shown to be effective in predicting functional outcome in AIS patients undergoing EVT (9). The relationship between CTTA based on hyperdense areas on post-thrombectomy NCCT images and FR after EVT remains unknown. Therefore, the aim of this study was to determine the role of CTTA in predicting FR after EVT in patients with PTIH and to construct and validate a model to predict FR specifically in patients with PTIH.

## 2 Materials and methods

### 2.1 Subjects

**Figure 1:**
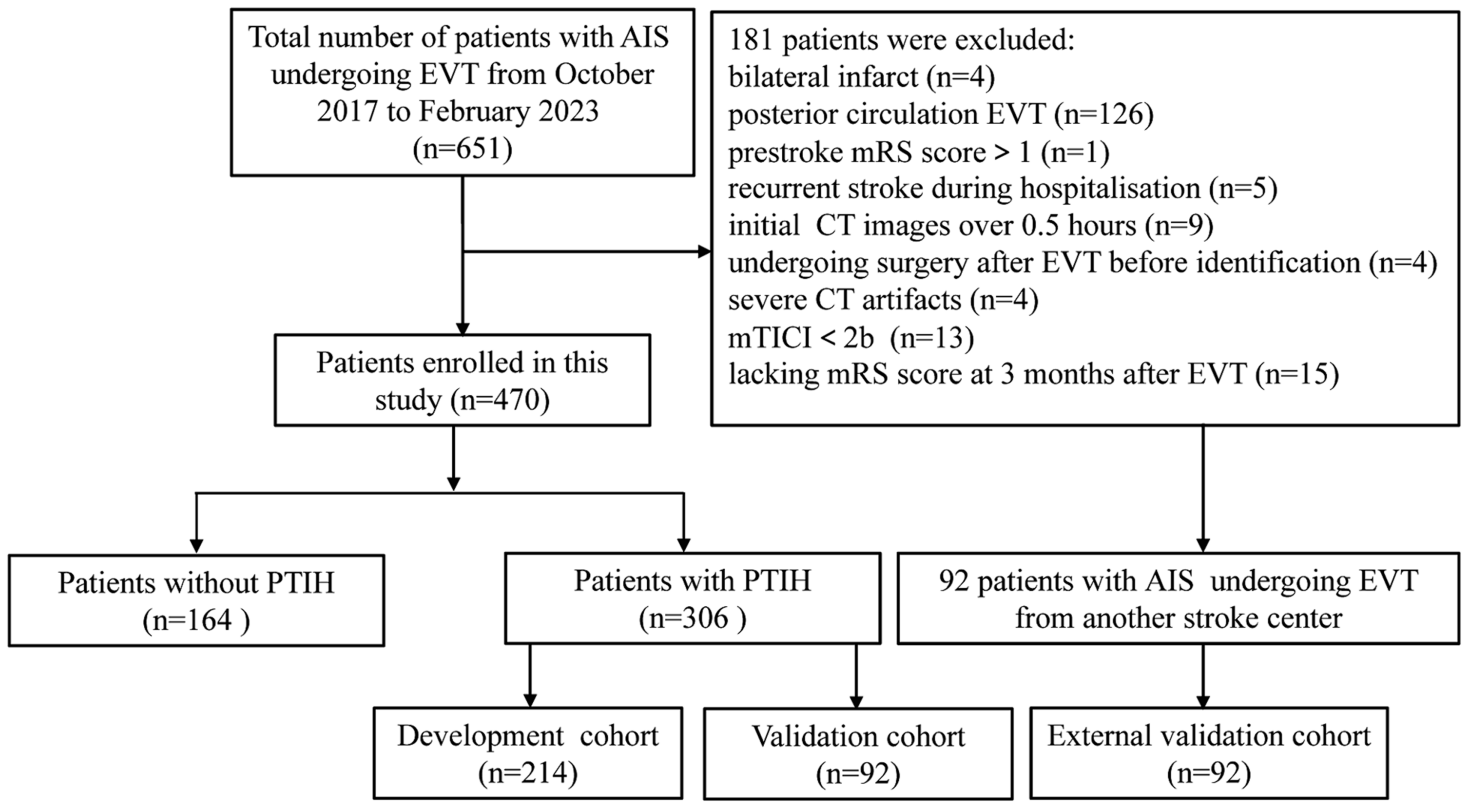
Flow chart of inclusion and exclusion criteria. AIS, acute ischemic stroke; EVT, endovascular thrombectomy; mTICI, modified thrombolysis in cerebral infarction score; mRS, modified Rankin scale; PTIH, post-thrombectomy intraparenchymal hyperdense.

The retrospective study was approved by the Ethics Committee of Huaian NO.1 People′s Hospital and the requirement for patient informed consent was waived (approval number KY-2023-046-01). A total of 651 patients with AIS underwent EVT at Huaian NO.1 People′s Hospital from October 2017 to February 2023. Inclusion criteria: (1) age ≥ 18 years; (2) patients of AIS with unilateral anterior circulation large artery occlusion undergoing EVT; (3) patients with successful reperfusion with modified thrombolysis in cerebral infarction score (mTICI) at level 2b-3; (4) patients with post-thrombectomy cranial NCCT images within 0.5 h of EVT; (5) patients with a prestroke mRS score of 0 to 1; (6) patients with mRS score at 3 months after EVT. Exclusion criteria: (1) patients with bilateral infarct; (2) patients with posterior circulation EVT; (3) patients with prestroke mRS score>1; (4) patients with recurrent stroke during hospitalization; (5) patients with initial CT images over 0.5 h; (6) patients undergoing surgery after EVT before identification; (7) patients with severe CT artifacts; (8) patients with mTICI < 2b; (9) patients lacking mRS score at 3 months after EVT. Patients with PTIH were finally randomized in a 7:3 ratio to the development and internal validation cohorts. 92 patients with PTIH from another stroke center (Xuzhou Medical University Affiliated Hospital of Huai’an) were assigned to the external validation cohort according to the same inclusion and exclusion criteria. The distribution diagram of the enrolled subjects in the training cohort, internal validation cohort and external validation cohort is shown in Figure 1.

### 2.2 Endovascular thrombectomy

All enrolled patients were selected according to stroke guidelines and underwent EVT under local anesthesia by two neurointerventionists with 10 years of experience. EVT was performed using stent retrievers [Solitaire AB (Covidien/ev3, Irvine, United States) and Solitaire FR (Covidien/ev3, Irvine, United States)] or react suction devices (Covidien/ev3, Irvine, United States). The physician performing the neurointervention regularly reported the number of stent retriever passes. If targeted arterial recanalisation failed, rescue therapies such as stent implantation, balloon angioplasty, intracatheter tirofiban administration or intra-arterial thrombolysis would be used.

### 2.3 Definition of PTIH and futile reperfusion

PTIH was defined as a new hyperdense area compared to surrounding brain tissue on NCCT images within 0.5 h after EVT. Good reperfusion was defined as patients who achieved successful reperfusion after EVT (mTICI at level 2b-3) with a favorable functional outcome at 3 months (mRS score ≤ 2). Futile reperfusion was defined as patients who achieved successful reperfusion after EVT but had an unfavorable outcome (mRS score ≥ 3).

### 2.4 Clinical data collection

The following baseline clinical data was collected, including demographic data (age and gender), vascular risk factors (smoking, drinking, atrial fibrillation, diabetes mellitus, hypertension, hyperlipidemia, coronary artery disease and previous stroke), systolic and diastolic blood pressure (SBP and DBP), baseline National Institutes of Health Stroke Scale (NIHSS) score, Alberta Stroke Program Early Computed Tomography (ASPECT) score, etiology of stroke (large-artery atherosclerosis, cardioembolism, stroke of other determined etiology and stroke of undetermined etiology), occlusion site (internal carotid artery and middle cerebral artery), endovascular therapy information (onset to door time, door to reperfusion time, onset to reperfusion time, cases treated with tirofiban, cases of thrombolysis, number of thrombectomy).

### 2.5 CT image acquisition and texture analysis

All CT images were acquired on the UCT 710 scanner. The scanning parameters were as follows: slice thickness: 5.0 mm, matrix size: 512 × 512, field of view: 21.1 × 21.1 cm, tube current: 199 mA, tube voltage: 120 kV. Finally, the scanned CT images are exported in Digital imaging and communications in medicine (DICOM) format.

ROI was manually segmented along the contour of the hyperdense area on the NCCT image by two experienced radiologists, using 3D-Slicer software (version 5.0.3). A total of 18 first-order histogram features were extracted from the ROI. Radiologists with 7 and 12 years of experience in diagnostic neuroradiology randomly selected 30 patients’ CT images and segmented them. The data were segmented twice with an interval of 1 week between segmentations by the radiologist with 7 years of experience. The data were then segmented again by a radiologist with 12 years of experience. The intraobserver and intergroup consistency of the ROI was tested using the intraclass correlation coefficient (ICC) (ICC > 0.8 indicates good agreement).

### 2.6 Statistical analysis

SPSS 24.0 software was used for statistical analysis. Continuous variables with normal distribution were expressed as mean ± S.E.M., and the two-tailed t-test was used to compare between different groups. Variables with skewed distribution were described by the median (interquartile range), and the Mann–Whitney U-test was used to compare differences between different groups. For categorical variables presented as frequency and percentage, the Chi-square test or Fisher’s exact test was used to analyze differences between groups. Univariate and multivariate logistic regression analysis was used to investigate the independent predictors of FR. No collinearity was assumed between variables included in the multivariate logistic regression if the tolerance (the tolerance range was 0–1) was greater than 0.1 and the variance inflation factor was less than 10. Multivariate logistic regression analysis estimated the odds ratio (OR) and 95% confidence interval (CI) for each variable. A *p* value <0.05 was considered statistically significant.

The nomogram was developed and validated using R software (version 4.2.3). Category-free net reclassification improvement (NRI) and integrated discrimination improvement (IDI) were used to compare the predictive power of the new and old models. The area under the receiver operating characteristic curve (AUC-ROC) was calculated to evaluate the discriminative ability of the nomogram. We calculated the positive predictive value (PPV), negative predictive value (NPV), and accuracy (ACC) of the model according to standard definitions. Calibration of the nomogram was performed using the Hosmer-Lemeshow test and a calibration plot with bootstraps of 1,000 resamples. Decision curve analysis (DCA) was used to evaluate the clinical net benefits of the nomogram.

## 3 Results

### 3.1 Baseline characteristics

**Figure 2:**
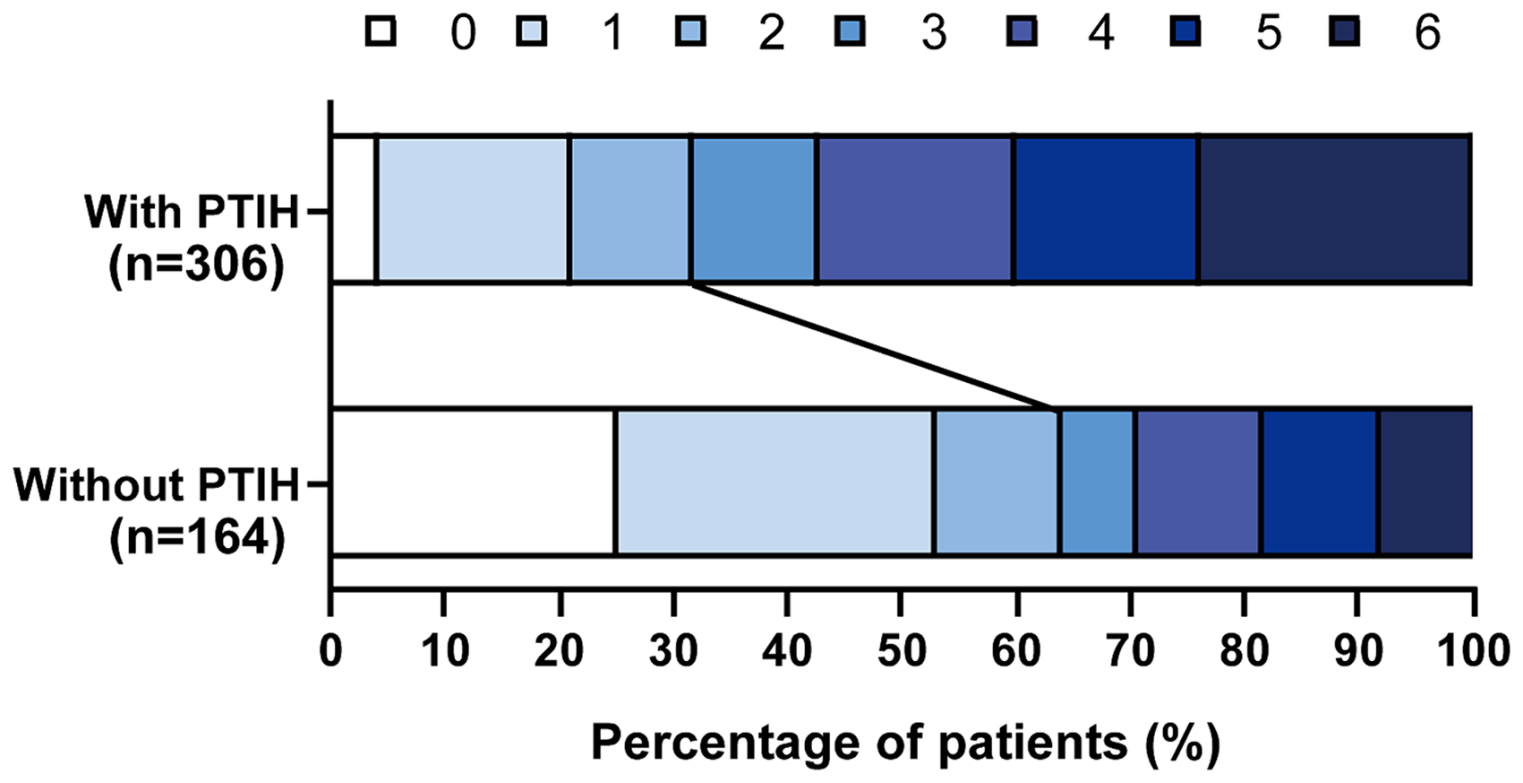
Distribution of the 3-month mRS scores for patients with and without PTIH.

A total of 651 patients with AIS underwent EVT from August 2017 to January 2023, and 181 patients were excluded according to the exclusion criteria (Figure 1). We analyzed the incidence of FR in 470 patients, including 306 patients with PTIH and 164 patients without PTIH. The results showed that the incidence of FR was significantly higher (*p* < 0.001) in patients with PTIH (68.3%) than in patients without PTIH (39.3%). In addition, the mortality was significantly higher (p < 0.001) in patients with PTIH (23.9%) than in patients without PTIH (7.9%). Of these deaths, 93% were stroke-related. The distribution of the 3-month mRS scores for patients with and without PTIH is shown in Figure 2.

Subsequently, 306 patients with PTIH were randomized in a 7:3 ratio to the development and internal validation cohorts, with 214 and 92 patients in the development and internal validation cohorts, respectively. 92 patients with PTIH from another stroke center were assigned to the external validation cohort. The baseline characteristics of the development and internal validation cohorts are shown in Table 1. The mean age of all patients was 69.26 ± 11.15 years and 164 (53.6%) patients were male. The incidence of FR was 68.3% (209 of 306) in all patients enrolled, 68.7% (147 of 214) in the development cohort, 67.4% (62 of 92) in the internal validation cohort.

**Table 1:** Comparison of baseline characteristics between the development cohort and internal validation cohort.

Clinical factors	All patients (*n* = 306)	Development cohort (*n* = 214)	Internal validation cohort (*n* = 92)	*p-*value
**Demographic data**				
Age (years)	69.26 ± 11.15	69.01 ± 11.18	69.89 ± 11.11	0.559^c^
male, *n* (%)	164 (53.6)	116 (54.2)	47 (51.1)	0.616^b^
**Vascular risk factors, *n* (%)**				
Diabetes mellitus	73 (23.9)	50 (23.4)	23 (25.0)	0.758^b^
Hypertension	191 (62.4)	130 (60.7)	61 (66.3)	0.357^b^
Hyperlipidemia	354 (11.1)	20 (9.3)	14 (15.2)	0.134^b^
Atrial fibrillation	176 (57.5)	121 (56.5)	56 (60.9)	0.482^b^
Coronary heart disease	31 (10.1)	22 (10.3)	9 (9.8)	0.895^b^
Previous stroke	21 (6.9)	15 (7.0)	6 (6.5)	0.877^b^
Smoking	69 (22.5)	48 (22.4)	21 (22.8)	0.939^b^
Drinking	68 (22.2)	47 (22.0)	21 (22.8)	0.869^b^
**Baseline data**				
SBP (mmHg)	149.78 ± 24.59	148.68 ± 24.74	152.36 ± 24.15	0.227^c^
DBP (mmHg)	84.93 ± 14.51	84.16 ± 14.75	86.71 ± 13.86	0.151^c^
NIHSS score	23 (18–26)	23 (18–26.25)	22 (18–26)	0.792^a^
ASPECT score	8 (7–9)	8 (7–9)	8 (7.25–9)	0.052^a^
**TOAST Classification, *n* (%)**		0.512^d^		
LAA	119 (38.9)	88 (41.1)	31 (33.7)	
CE	180 (58.8)	121 (56.5)	59 (64.1)	
SOE	6 (2)	2 (1.9)	2 (2.2)	
SUE	1 (0.3)	1 (0.5)	0 (0)	
**Occlusion site, *n* (%)**				0.169^b^
ICA	138 (45.1)	102 (47.7)	36 (39.1)	
MCA	168 (54.9)	112 (52.3)	56 (60.9)	
**Endovascular therapy**				
Tirofiban, *n* (%)	79 (25.8)	58 (27.1)	21 (22.8)	0.433^b^
Thrombolysis, *n* (%)	70 (22.9)	51 (23.8)	19 (20.7)	0.544^b^
Number of thrombectomy	2 (1–3)	2 (1–3)	2 (1–3)	0.733^a^
ODT (min)	209.56 ± 101.29	212.90 ± 99.73	201.79 ± 104.98	0.390^c^
DRT (min)	166 (125–207)	166 (126.5–211.25)	166 (121.50–204.75)	0.812^a^
ORT (min)	386.02 ± 113.62	391.59 ± 114.64	376.07 ± 111.07	0.268^c^
Futile reperfusion, *n* (%)	209 (68.3)	147 (68.7)	62 (67.4)	0.823 ^b^

Values are listed as frequency (percentage), mean ± SD or median (interquartile range). *P*-value represents the comparison between the development cohort and validation cohort, with “a” representing Mann–Whitney U-test, “b” representing Chi-square test, “c” representing Student’s t-test, and “d” representing Fisher’s exact test. SBP, systolic blood pressure; DBP, diastolic blood pressure; NIHSS, National Institute of Health stroke scale; ASPECT, Alberta Stroke Program Early Computed Tomography Score; TOAST, Trial of Org 10,172 in Acute Stroke Treatment; LAA, large-artery atherosclerosis; CE, cardioembolism; SOE, stroke of other determined etiology; SUE, stroke of undetermined etiology; ICA, internal carotid artery; MCA, Middle cerebral artery; ODT, onset to door time; DRT, door to reperfusion time; ORT, onset to reperfusion time.

### 3.2 Predictors for FR in patients with PTIH

We first screened for clinical predictors for FR in patients with PTIH. There were significant differences between the two groups in age, NIHSS score, ASPECT score, door to reperfusion time (DRT) and onset to reperfusion time (ORT), as shown in Table 2. These five variables were further analyzed with univariate analysis, and the results revealed that age, NIHSS score, ASPECT score and ORT were associated with FR (Table 3). Similarly, we then analyzed for CT texture features extracted from hyperdense areas on NCCT images within half an hour after EVT. A total of 14 of the 18 first-order histogram features were significantly different between the two groups (Table 4), and these were further analyzed using univariate analysis. 12 features were found to be associated with FR (Table 3). Finally, age (OR: 1.045, 95% CI: 1.006–1.086), NIHSS score (OR: 1.194, 95% CI: 1.103–1.292), ASPECT score (OR: 0.459, 95% CI: 0.322–0.654), entropy (OR: 2.321, 95% CI: 1.104–4.879) and kurtosis (OR: 1.492 95% CI: 1.100–2.024) were independently related to FR in the multivariate logistic regression analysis, with ASPECT score being a protective factor, whereas NIHSS score, entropy and kurtosis were risk factors (Table 3).

**Table 2:** Comparison of baseline characteristics of patients with futile and good reperfusion in the development cohort.

Clinical factors	Good reperfusion (*n* = 67)	Futile reperfusion (*n* = 147)	*p-*value
**Demographic data**			
Age (years)	66.66 ± 11.79	70.09 ± 10.76	0.045^c^
male, *n* (%)	41 (61.2)	78 (53.1)	0.267^b^
**Vascular risk factors, *n* (%)**			
Diabetes mellitus	16 (23.9)	34 (23.1)	0.904^b^
Hypertension	42 (62.7)	88 (59.9)	0.695^b^
Hyperlipidemia	6 (9.0)	14 (9.5)	0.895^b^
Atrial fibrillation	39 (58.2)	82 (55.8)	0.740^b^
Coronary heart disease	6 (9.0)	16 (10.9)	0.667^b^
Previous stroke	5 (7.5)	10 (6.8)	0.861^b^
Smoking	16 (23.9)	32 (21.8)	0.731^b^
Drinking	17 (25.4)	30 (20.4)	0.416^b^
**Baseline data**			
SBP (mmHg)	148.15 ± 24.87	148.92 ± 24.77	0.834^c^
DBP (mmHg)	85.90 ± 15.50	83.37 ± 14.38	0.261^c^
NIHSS score	19 (15–21)	26 (22–28)	<0.001^a^
ASPECT score	9 (8–10)	8 (6–8)	<0.001^a^
**TOAST classification *n* (%)**			0.325^d^
LAA	34 (44.2)	24 (33.8)	
CE	41 (53.2)	46 (64.8)	
SOE	2 (2.6)	0 (0)	
SUE	0 (0)	1 (1.4)	
**Occlusion site, *n* (%)**			0.145^b^
ICA	27 (40.3)	75 (51)	
MCA	40 (59.7)	72 (49)	
**Endovascular therapy**			
Tirofiban, *n* (%)	22 (32.8)	36 (24.5)	0.203^b^
Thrombolysis, *n* (%)	17 (25.4)	34 (23.1)	0.721^b^
Number of thrombectomy	2 (1–3)	2 (1–3)	0.111^a^
ODT (min)	196.40 ± 90.07	220.41 ± 103.26	0.087^c^
DRT (min)	150.00 (120–200)	176 (130–219)	0.030^a^
ORT (min)	360.84 ± 111.98	405.61 ± 113.46	0.008^c^

Values are listed as frequency (percentage), mean ± SD or median (interquartile range). *P*-value of “a” represents Mann–Whitney U-test, “b” represents Chi-square test, “c” represents Student’s *t*-test, and “d” represents Fisher’s exact test. SBP, systolic blood pressure; DBP, diastolic blood pressure; NIHSS, National Institute of Health stroke scale; ASPECT, Alberta Stroke Program Early Computed Tomography Score; TOAST, Trial of Org 10,172 in Acute Stroke Treatment; LAA, large-artery atherosclerosis; CE, cardioembolism; SOE, stroke of other determined etiology; SUE, stroke of undetermined etiology; ICA, internal carotid artery; MCA, Middle cerebral artery; ODT, onset to door time; DRT, door to reperfusion time; ORT, onset to reperfusion time.

**Table 3:** Logistic regression analysis for predictors of futile reperfusion in patients with PTIH in the development cohort.

Variables	Univariate analysis		Multivariate analysis	
	OR (95%CI)	*p-*value	OR (95%CI)	*p-*value
Age (years)	1.028 (1.001–1.055)	0.039	1.045 (1.006–1.086)	0.023
NIHSS score	1.245 (1.163–1.334)	<0.001	1.194 (1.103–1.292)	<0.001
ASPECT score	0.410 (0.304–0.554)	<0.001	0.459 (0.322–0.654)	<0.001
DRT (min)	1.004 (0.999–1.008)	0.106		
ORT (min)	1.004 (1.001–1.007)	0.009		
Interquartile range	1.018 (0.999–1.038)	0.060		
90 percentile	1.010 (1.001–1.020)	0.004		
Median	1.023 (1.002–1.045)	0.033		
Uniformity	0.041 (0.006–0.295)	0.002		
Robust mean absolute deviation	1.049 (1.000–1.099)	0.048		
Mean absolute deviation	1.036 (1.002–1.070)	0.036		
Total Energy	1.000 (1.000–1.000)	0.016		
Maximum	1.005 (1.001–1.009)	0.010		
Minimum	0.985 (0.961–1.009)	0.213		
Entropy	4.950 (2.508–9.768)	<0.001	2.321 (1.104–4.879)	0.026
Range	1.005 (1.001–1.008)	0.008		
Variance	1.002 (1.001–1.004)	0.002		
Kurtosis	1.518 (1.197–1.926)	0.001	1.492 (1.100–2.024)	0.010
Root mean squared	1.020 (1.003–1.037)	0.018		

NIHSS, National Institute of Health stroke scale; ASPECT, Alberta Stroke Program Early Computed Tomography Score; DRT, door to reperfusion time; ORT, onset to reperfusion time.

**Table 4:** Comparison of CT texture features in patients with futile and good reperfusion in the development cohort.

CT texture	Good reperfusion (*n* = 67)	Futile reperfusion (*n* = 147)	*p-*value
Interquartile range	16.50 (12.00–25.25)	20 (14.00–32.00)	0.020^a^
10 Percentile	39.01 ± 5.4	39.65 ± 7.16	0.747^b^
90 Percentile	68 (60–87.5)	80 (63–99)	0.009^a^
Median	51 (46–64)	56 (47–68)	0.032^a^
Uniformity	0.48 ± 0.15	0.40 ± 0.15	0.001^b^
Skewness	0.32 (0.11–0.79)	0.40 (0.19–0.91)	0.329^a^
Energy (×10^7^)	0.42 (0.06–1.1)	0.49 (0.11–1.97)	0.143^a^
Robust mean absolute deviation	7.19 (5.59–11.16)	8.49 (6.29–13.61)	0.018^a^
Mean absolute deviation	9.84 (7.54–14.59)	12.18 (8.93–18.76)	0.006^a^
Total energy (×10^7^)	1.61 (0.66–5.48)	5.39 (0.17–14.23)	<0.001^a^
Maximum	89 (80–129)	118 (86–209)	<0.001^a^
Minimum	24 (21–27)	22 (18–26)	0.046^a^
Mean	54.33 (47.75–67.23)	60.12 (49.23–71.98)	0.083^a^
Entropy	1.15 (1.01–1.38)	1.70 (1.27–2.06)	<0.001^a^
Range	69 (55–98)	98 (65–220)	<0.001^a^
Variance	164.23 (111.85–231.42)	251.63 (175.10–504.98)	<0.001^a^
Kurtosis	2.76 (2.28–3.61)	3.06 (2.44–5.23)	0.001^a^
Root mean squared	52.12 (47.70–64.46)	62.23 (49.85–75.95)	0.004^a^

Values are listed as mean ± SD or median (interquartile range). *P*-value of “a” represents Mann–Whitney U-test and “b” represents Student’s *t*-test.

### 3.3 Development of the nomogram to predict FR in patients with PTIH

**Figure 3:**
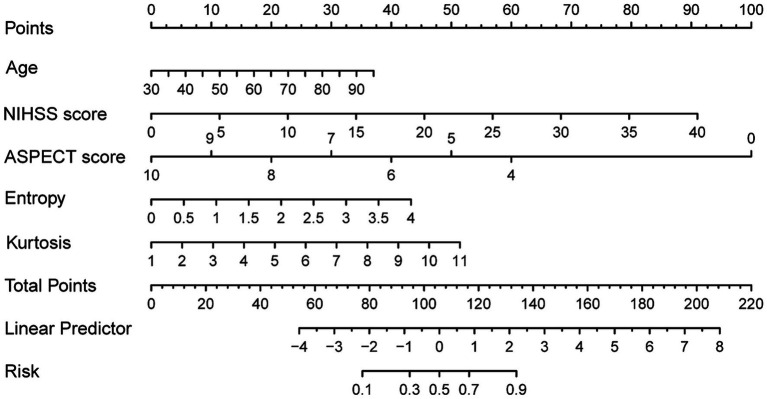
Nomogram to predict futile reperfusion in patients with PTIH.

The following five independent predictors of FR based on the results of multivariate logistic regression: age, NIHSS score, ASPECT score, entropy and kurtosis, were used to construct the nomogram for predicting FR in patients with PTIH (Figure 3). The nomogram, which is a graphical statistical tool that can calculate and estimate the probability of clinical outcome for individuals using a continuous score, consists of a series of higher and lower scoring line segments representing the contribution of predictors to FR, with each predictor being assigned a specific value based on the scale on the line segment. The total score is obtained by summing the scores of each predictor. Finally, a vertical line is drawn from the total score line to the risk line. The risk corresponding to the total score represents the estimated probability of FR. For example, a patient with PTIH aged 83 has a baseline NIHSS score of 22, ASPECT score of 10, entropy of 1.90 and kurtosis of 2.41. The point corresponding vertically to each variable is 30 for age, 50 for NIHSS score, 0 for ASPECT score, 20 for entropy and 8 for kurtosis. The total point for this patient is 108, corresponding to 0.52 on the risk line, indicating a 52% probability of FR for this patient.

### 3.4 Validation of the nomogram

To compare the predictive power of the novel nomogram (combining CT textures and clinical factors) and the old clinical model (including clinical factors only), we analyzed the category-free NRI and IDI. The category-free NRI was 0.204 (95% CI: 0.073–0.334, *p* = 0.002) in the development cohort, 0.316 (95% CI: 0.090–0.542, *p* = 0.006) in the internal validation cohort and 0.458 (95% CI: 0.247–0.670, *p* < 0.001) in the external validation cohort. The IDI was 0.063 (*p* < 0.001) in the development cohort, 0.105 (*p* = 0.003) in the internal validation cohort and 0.170 (*p* < 0.001) in the external validation cohort. These results demonstrated the superior predictive power of the novel nomogram combining CT textures and clinical factors over the old clinical model.

**Figure 4:**
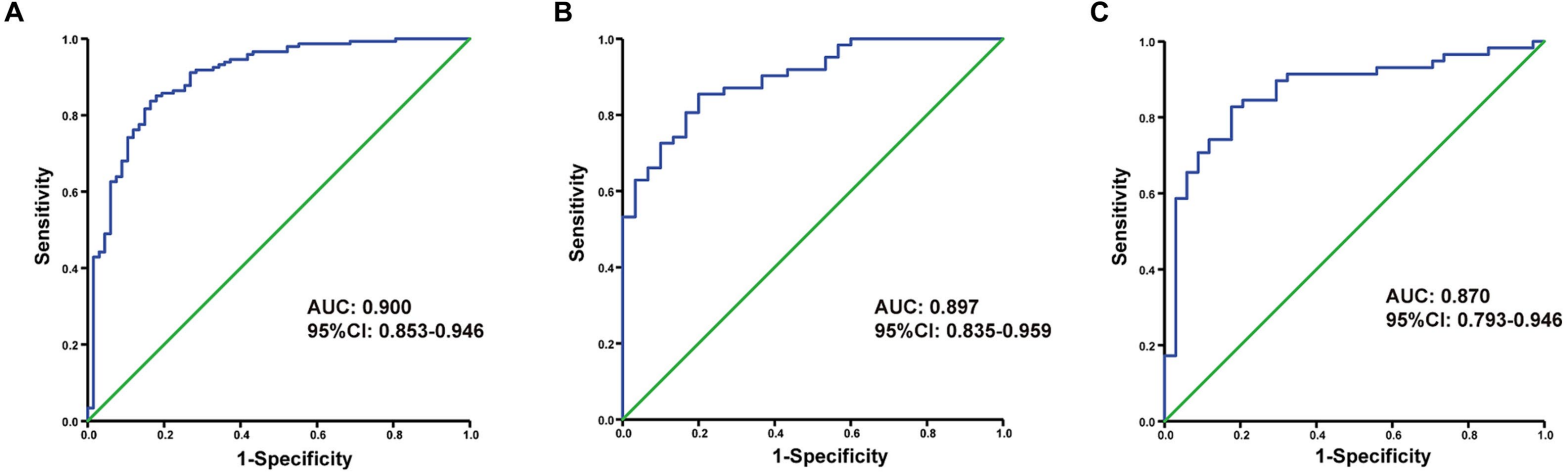
The ROC curve of the nomogram for predicting futile reperfusion in patients with PTIH in the development **(A)**, internal validation **(B)** and external validation **(C)** cohorts.

**Figure 5:**
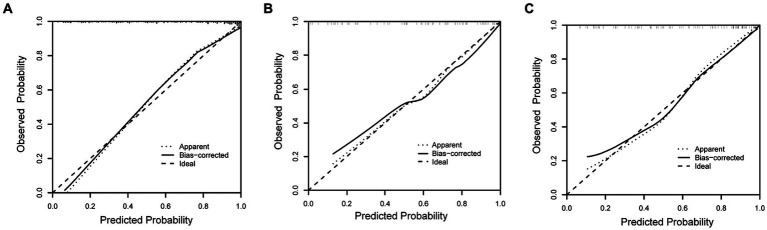
Calibration plot for predicting futile reperfusion in patients with PTIH in the development **(A)**, internal validation **(B)** and external validation **(C)** cohorts.

**Figure 6:**
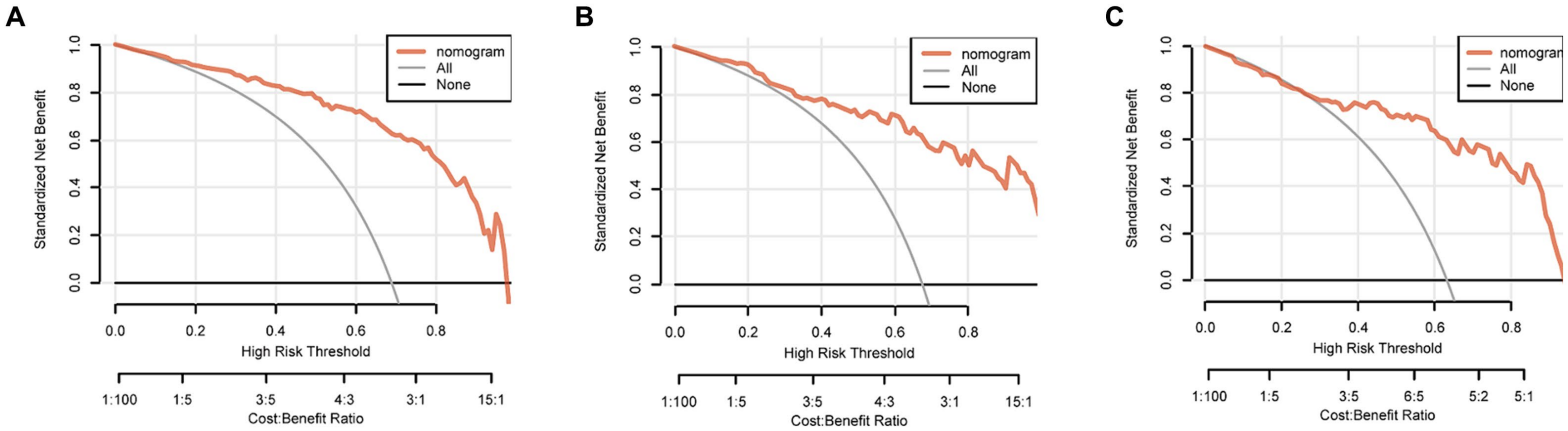
Decision curve of the nomogram predicting futile reperfusion in patients with PTIH in the development **(A)**, internal validation **(B)** and external validation **(C)** cohorts.

The discriminating performance of the nomogram was assessed by the area under the receiver operating characteristic curve (AUC-ROC) in the development and validation cohorts. The AUC-ROC was 0.900 (95% CI: 0.853–0.946) in the development cohort, 0.897 (95% CI: 0.835–0.959) in the internal validation cohort and 0.870 (95% CI: 0.793–0.946) in the external validation cohort, which indicating moderate predictive power (Figures 4A–C). In addition, for the training, internal validation, and external validation cohorts, at the best threshold, the PPV was 91.8, 87.9, and 88.9%, respectively; the NPV was 70.0%, 76.4%, and 73.7%, respectively; and the ACC was 83.7, 83.5, and 82.7%, respectively. The Hosmer-Lemeshow test indicated high goodness of fit between predicted and observed probability for the development cohort (χ^2^ = 12.121, df = 8, *p* = 0.146), the internal validation cohort (χ^2^ = 4.877, df = 8, *p* = 0.771) and the external validation cohort (χ^2^ = 10.017, df = 8, *p* = 0.264). The calibration plot, with the x-axis representing the probability of the predicted outcome and the y-axis representing the probability of the outcome actually occurring, revealed good consistency between the predicted and the actual outcomes (Figures 5A–C). The DCA indicated good positive net benefit and clinical validity of the nomogram (Figures 6A–C), as the range of threshold probabilities was wide and practical.

## 4 Discussion

In this study, we constructed and validated a nomogram which combining clinical factors and CT texture features extracted from hyperdense areas on CT images within half an hour after EVT, to predict FR in patients with PTIH. Notably, the novel nomogram based on five predictors, including age, NIHSS score, ASPECT score, entropy and kurtosis, allowed clinicians to stratify the risk of FR in patients with PTIH.

Currently, FR is becoming a major challenge in the treatment of patients with AIS by mechanical thrombectomy (3). However, the pathophysiology of FR remains unclear. The underlying mechanisms of FR include the “no-reflow” phenomenon, initial tissue damage, reperfusion injury, cerebral edema, poor collateral flow and inflammation (3, 10). Notably, reperfusion injury is an important mechanism of FR and many studies have identified it as an independent risk factor for poor prognosis 3 months after EVT (11, 12). Previous studies have shown that the occurrence of PTIH is strongly associated with reperfusion injury after EVT (12, 13), which may be one of the mechanisms for the higher rate of FR in patients with PTIH (68.3% in our study) than in those without PTIH (39.3% in our study). Therefore, it will be of great clinical value to stratify the risk of FR specifically in patients with PTIH by developing predictive nomogram models.

PTIH, representing intracerebral hemorrhage or contrast extravasation, is an important radiographic feature of cerebral reperfusion injury after EVT (12, 13). Although intracerebral hemorrhage is a known and important risk factor for FR (3, 10), identification of whether the hyperdense area on CT images is hemorrhage or contrast leakage is based on follow-up CT 24 h after EVT, which may delay treatment adjustment. It has been reported that quantitative analysis of hematoma texture features on cranial CT may contribute to improved prediction of clinical outcome in symptomatic intracerebral hemorrhage (14). In our study, we demonstrated that CT textures extracted from hyperdense areas on NCCT images in patients with PTIH were independent predictors of FR. CT texture features can be divided into first-order and second-order features. First-order features are based on histogram analysis and represent the distribution of pixel values per gray level (15). Second-order features provide results on the spatial formation of voxels by calculating statistical relationships between neighboring voxels (16). First-order histogram features are more accessible than second-order features and are just as informative. In our study we extracted a total of 18 first-order features from hyperdense areas on NCCT images. Finally, entropy and kurtosis were identified as independent predictors for FR in patients with PTIH.

To our knowledge, our study is the first to construct a nomogram based on CTTA to predict FR in patients with PTIH. Our results showed that higher entropy and kurtosis were associated with a higher risk of FR. Sarioglu et al. (9) found that CT textures were effective in predicting unfavorable functional outcome in AIS patients undergoing EVT. In contrast to our study, they extracted CT texture features not from hyperdense areas on NCCT images in patients with PTIH, but from early ischemic CT signs. Kanazawa et al. (17) reported that the mean CT value of clots in the subarachnoid space could predict clinical outcome in patients with subarachnoid hemorrhage, whereas we did not find the predictive role of the mean CT value.

There are several limitations in the present study. First, it was a single-center retrospective study which may have some selection bias, and further multicenter prospective studies are needed to reduce bias. Second, ROIs were drawn by hand and based on the experience of observers. We attempted to address this issue by assessing the repeatability of two independent observers. Third, we did not extract second-order features, which are informative but relatively difficult to extract and analyze. More comprehensive studies of CTTA are needed to further analyze the association between CTTA and FR in patients with PTIH. Finally, the sample size of the study was small and large sample studies are needed to confirm our findings.

In conclusion, we developed and validated a nomogram based on clinical factors and CT texture features extracted from hyperdense areas on CT images within half an hour after EVT to predict FR in patients with PTIH. The proposed nomogram was able to accurately stratify the risk of FR specifically in patients with PTIH. It may help clinicians to formulate more appropriate treatment plans for high-risk patients in the early post-EVT period and has clinical promotional value. In addition to predicting FR, CT texture is valuable in other aspects of stroke, including the diagnosis of stroke lesions (18–20) and cerebral hemorrhage (21, 22). In the future, we should intensify our research on CT texture analysis to fully unveil its value and expand its application.

## Data availability statement

The original contributions presented in the study are included in the article/supplementary material, further inquiries can be directed to the corresponding author.

## Ethics statement

The studies involving humans were approved by the Ethics Committee of Huaian No.1 People′s Hospital. The studies were conducted in accordance with the local legislation and institutional requirements. The ethics committee/institutional review board waived the requirement of written informed consent for participation from the participants or the participants’ legal guardians/next of kin because this a retrospective study and the requirement for patient informed consent was waived.

## Author contributions

MD: Writing – original draft, Validation, Project administration, Methodology, Investigation, Formal analysis, Data curation, Conceptualization. CC: Writing – review & editing, Writing – original draft, Validation, Methodology, Formal analysis, Data curation, Conceptualization. WC: Writing – original draft, Validation, Software, Methodology, Formal analysis, Data curation. KA: Writing – review & editing, Writing – original draft, Supervision, Project administration, Data curation, Conceptualization.
